# Effects of Inlet Geometry on Perfusion and Ischemia in Anomalous Aortic Origin of the Right Coronary Artery (AAORCA)

**DOI:** 10.7759/cureus.76579

**Published:** 2024-12-29

**Authors:** Ryan DeGroff, Dalia Lopez-Colon, Arun Chandran, Diego Moguillansky, Curt DeGroff

**Affiliations:** 1 Department of Pediatrics, University of Cincinnati College of Medicine, Cincinnati, USA; 2 University of Florida Health Congenital Heart Center, University of Florida College of Medicine, Gainesville, USA; 3 Department of Cardiovascular Medicine-Internal Medicine, University of Florida Health Congenital Heart Center, University of Florida College of Medicine, Gainesville, USA

**Keywords:** anomalous aortic origin of a coronary artery, cardiology, computational, coronary ischemia, simulations

## Abstract

Anomalous aortic origin of a coronary artery (AAOCA) comprises a set of rare congenital abnormalities in the origin or path of the coronary arteries with highly variable clinical implications. This is a pilot feasibility study where we investigated the influence of the anomalous coronary artery inlet architecture on coronary perfusion using coronary blood flow computational simulations to help predict the risk for coronary ischemia in patients with anomalous aortic origin of the right coronary artery (AAORCA) with these types of anomalous coronary artery inlet architectures. We developed a protocol for generating 3D models of patient coronary artery anatomies from an IRB-approved dataset of cardiac CT images of patients with AAORCA at our institution. Coronary blood flow simulations and analysis were performed. Instantaneous flow reserve (iFR), a parameter used clinically in coronary CT analysis to determine risk for ischemia, was compared between models as a measure of ischemia. Comparing the median iFR of the coronary outlets between the four inlet variants and baseline architecture showed important differences. We observed a possible association between the proportion of the semi-minor axis and the semi-major axis of the elliptical AAORCA inlet and iFR. These observations suggest that the elliptical axis quotient may be a significant risk factor for evaluation of AAORCA severity.

## Introduction

Anomalous aortic origin of a coronary artery (AAOCA) comprises a set of rare congenital abnormalities having anomalous origin and anomalous pathway of a coronary artery with highly variable clinical implications [1-6]. AAOCA is the second leading cause of sudden cardiac death (SCD) in young athletes [7]. The decision to pursue surgical management to reduce the risk of SCD in asymptomatic AAOCA patients, particularly in patients with the subset of anomalous aortic origin of the right coronary artery (AAORCA), remains a hotly debated topic due to the lack of quantitative risk factors predictive of future cardiac ischemia without surgery [8,9].

This is a pilot feasibility study where we use open-source code computational modeling to investigate the influence of the anomalous coronary artery inlet orifice architecture on coronary perfusion to help in predicting the risk for coronary ischemia in patients with AAORCA. Computational modeling allows for modeling of complex structures with significant anatomic and physiologic accuracy and readily allows for minor to major modifications to any of the models developed.

Other investigators have studied the effects of variations in anatomical features of AAORCA such as the length of a transmural segment and acute angle of coronary takeoff [10,11]. We studied AAORCA models differing in minor-to-major axis orifice ratios all with equal orifice cross-sectional areas to see if there are differences in ischemic risk with changes to minor-to-major axis orifice architectures. Perhaps what is presented here will lead to further study where a scoring system for assessing ischemic risk in patients with AAORCA is developed with differences in minor-to-major axis orifice architectures being one of several criteria used to develop an ischemic risk score. The criteria we propose here will require computational modeling for each patient evaluated. We would expect having a scoring system will help potentially determine which patients require intervention and who can be watched more conservatively.

## Materials and methods

**Figure 1:**
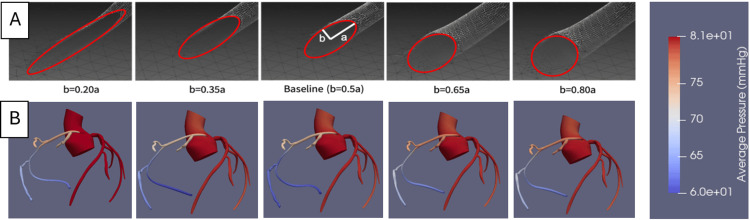
A. Overlay of elliptical inlet geometry on SimVascular models. B. Heatmap of average simulated pressures in the aorta and coronary tree.

Models were built using CT images which is the gold standard for imaging patients with AAOCA [12-14]. We developed a protocol for generating 3D models of patient coronary artery anatomies from an IRB-approved dataset of cardiac CT images of patients with AAORCA at our institution (University of Florida, Gainesville). Coronary blood flow simulations and analysis were then performed using a popular open-source computational fluid dynamic software package SimVascular [15]. For this feasibility study, we developed one patient’s baseline AAORCA anatomy and then four elliptical inlet coronary variant models of equal cross-sectional area were generated within a clinically relevant range of major-to-minor elliptical axis ratios (Figure 1A) [16].

Cardiac cycle simulations were run on each of these models. Simulations of six consecutive cardiac cycles for each model were run. After verifying adequate convergence of simulation results, instantaneous flow reserve (iFR), a parameter used clinically in coronary CT analysis to determine risk for ischemia, was compared between models as a measure of ischemia.

Using the patient’s native anatomy, the proportion (𝐶) of the measured semi-minor axis (𝑏) to the semi-major axis (𝑎) of the patient’s native AAORCA elliptical inlet was calculated, where 𝐶 = b/a. Four inlet variants with equivalent area and varying values of 𝐶 were created such that the orifice area is given by 𝐴 = 𝜋∗𝑎∗𝑏 and the range of 𝐶 is given by 𝐶native+/- 0.30. These variant dimensions represent a similar range of clinically observed interarterial elliptical AAORCA orifice height-to-width ratios outlined in the current literature [16].

Meshes of 800,000 elements were created for each model. We chose this mesh size based on similar coronary models in SimVascular [15]. Boundary conditions were set using patient-specific values (mean arterial pressure from the blood pressure cuff device, pulse from echocardiogram - in this case 60 bpm, and stroke volume estimated from the patient’s echocardiogram) found on or recent to the date of the patient’s cardiac CT study. Simulations of 6000 milliseconds are then run on each model. Adequate convergence was assessed by comparing systolic and diastolic pressures in the aortic pressure waveform for the final 1000 milliseconds to patient aortic values. Values of the iFR for each model are calculated by dividing the outlet pressures of the AAORCA branch by the aortic pressure over the diastolic interval. Due to the current limited sample size and the subsequent limitations of assuming a normal distribution of data, nonparametric statistical analysis was conducted on the median iFR between models using Minitab® (Minitab LLC, State College, PA) [17].

## Results

Simulations of coronary flow solutions for each model were successful (Figure 1B). The Kruskal-Wallis one-way ANOVA hypothesis test [18] for differences in the median iFR between all groups showed that at least one group differed statistically significantly (p <0.001). A reduction in the b/a quotient by 0.30 (b=0.2a vs. baseline) is associated with a clinically significant reduction in the median iFR below the clinical 0.8 threshold for ischemia.

The sign test tests if the median of a collection is clinically significantly greater than or less than a specific value, in this case 0.8 iFR. A sign test for a median 0.8 iFR was conducted to compare each group to the iFR < 0.8 clinical threshold for ischemia. The b=0.20a model was shown to have clinically significant reduction in the median iFR below the 0.8 threshold (p = 0.007). Mann-Whitney U tests [19] comparing the median iFR of the coronary outlets between the four inlet variants and baseline architecture showed a statistically significantly reduced iFR for the variant inlet with a semi-minor axis equal to 20% the length of the semi-major axis (p < 0.001 when compared to the baseline). The reduction meets the iFR ≤ 0.8 clinical threshold for coronary ischemia. A statistically significant increase was found in the iFR for both the b=0.65a and b=0.80a variants (p = 0.001, p < 0.001, respectively). This suggests that certain elliptical inlet orifice shapes are more and some are less likely to cause ischemia than the baseline architecture.

**Figure 2:**
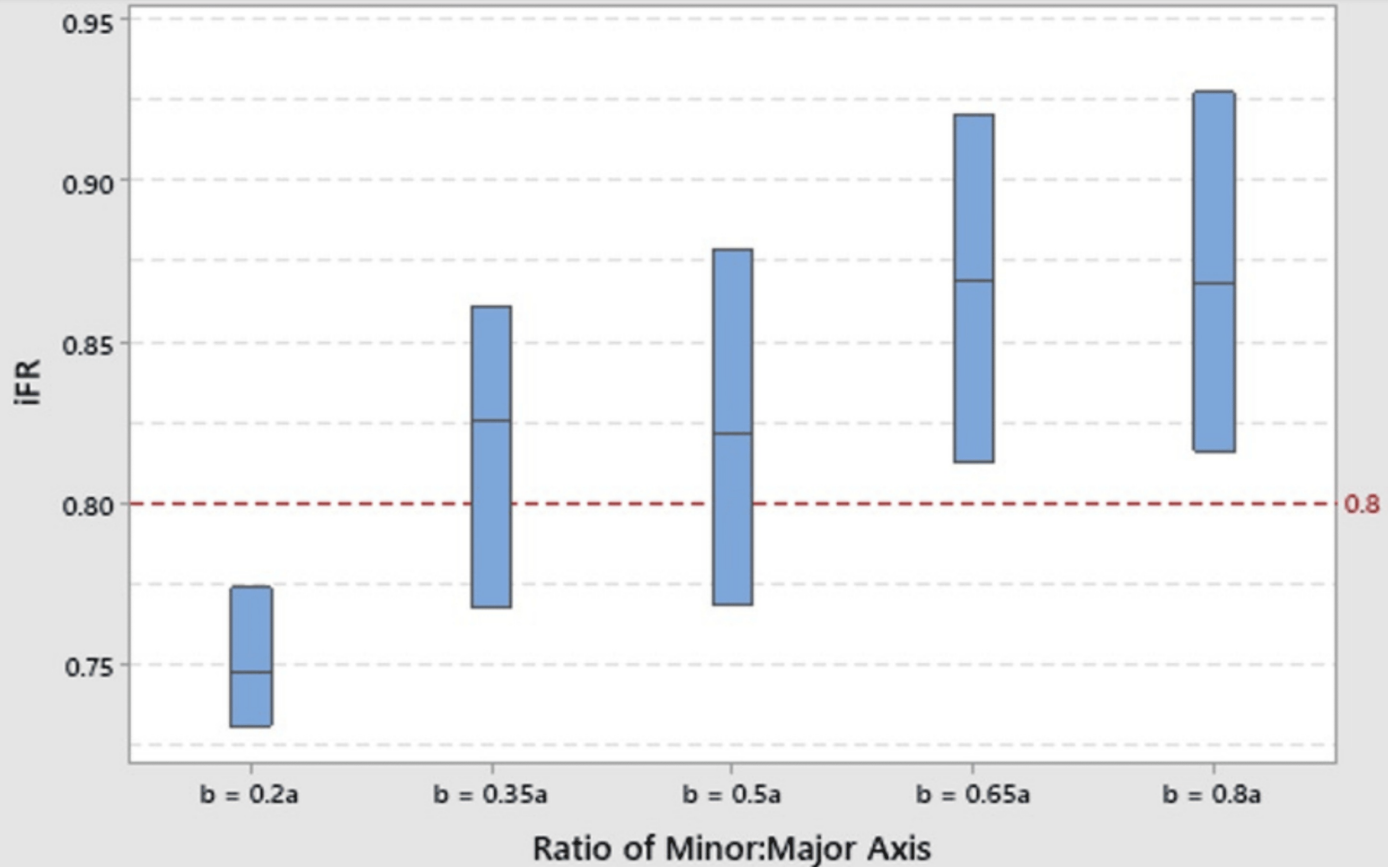
Boxplot of median confidence intervals of iFR for the baseline model (b=0.5a) and for the variant models. iFR: Instantaneous flow reserve

A box plot of the median iFR confidence intervals for each model is shown in Figure 2. This box plot conveys the distribution of the data within each dataset highlighting the median, quartiles and potential outliers.

## Discussion

Decades of investigations using computational modeling to study the cardiovascular system have led to a deeper understanding of the functionality of numerous parts of the cardiovascular system and in many applications, this is considered a valuable tool for clinical decision making. Computational methodologies have been used to predict the extent of coronary artery stenosis [20] and have shown promise in personalized surgical bypass coronary planning [21].

AAORCA has some similarities but also has some important differences from coronary artery stenosis physiology [22,23]. Investigators have used modeling studies to investigate the effects of variations in anatomical features of AAORCA like the length of transmural segment, acute angle of coronary takeoff, and slit-like versus normal arterial lumen on parameters of blood perfusion through computational modeling and in-vitro experimentation to help assess the relative risk of AAORCA variants [10,11]. The reduction in the iFR (a popular parameter used clinically to determine risk of coronary ischemia and thereby pressure drop) that is seen in these slit-like luminal studies when compared to normal coronary orifice anatomy is likely largely due to a relative reduction in the cross-sectional surface area at the coronary orifice. However, the effects on the iFR on varying coronary orifice shapes in models with coronary orifices having equal cross sectional orifice area are less well studied. The shape of coronary orifices with similar coronary orifice cross-sectional areas may become an important factor in itself in assessing the ischemic risk in patients with AAORCA. Findings from this pilot feasibility study show that differences do exist between models with different coronary orifice architectures that have the same orifice cross sectional area.

There are a number of limitations to this study. For this pilot feasibility study, we developed one patient’s baseline AAORCA anatomy for our baseline computational model and then created four elliptical inlet coronary artery orifice variant models of equal coronary orifice cross-sectional area varying the minor-to-major axis ratio. We plan on investigating the predictive value of this minor-to-major axis ratio with additional boundary conditions (e.g., stress test boundary conditions) as well as models derived from images from other patients with AAORCA to see if a scoring system for assessing ischemic risk in all patients with AAORCA can be developed with differences in minor-to-major axis orifice architectures being one of several criteria used to develop an ischemic risk score.

## Conclusions

From nonparametric statistical analysis of our pilot feasibility study, we observed an association between the proportion of the minor axis to the major axis of the elliptical AAORCA inlet and iFR, a parameter used clinically in coronary CT analysis to determine risk for ischemia. These observations suggest that this elliptical axis quotient may be a significant risk factor for the evaluation of AAORCA ischemic severity.
